# Are Oral Implants the Same As Teeth?

**DOI:** 10.3390/jcm8091501

**Published:** 2019-09-19

**Authors:** Tomas Albrektsson

**Affiliations:** Department of Biomaterials, University of Gothenburg, 40530 Gothenburg, Sweden; tomas.albrektsson@biomaterials.gu.se; Tel.: +46-705916607

Osseointegration of oral implants was initially discovered by Brånemark. The time for his discovery has incorrectly been said to have been during the 1950s [1,2], but in reality, the year was 1962 [3]. Brånemark operated the first patient with oral implants in 1965, only three years after his discovery. Osseointegration has meant a breakthrough for clinical results in oral and craniofacial implants [4,5] and has been applied, if in relatively small numbers, for anchorage of orthopedic implants in amputees [6]. The original definition of the term implied a direct contact, at the light microscopic level of resolution, between bone tissue and load-bearing implants [7,8]. Hip and knee arthroplasties do not present a direct bone anchorage, but instead display distance osteogenesis [9], probably due to the substantial clinical trauma at insertion. Nevertheless, clinical results of orthopedic implants have remained quite good, with 88% of operated hip replacements still in situ 25 years after surgery [10].

In 1985, I started seeing a researcher who later became a good friend. “Tomas” said this man to me in German, you must realize that osseointegration is but a “fremderkörperreaktion”—a foreign body reaction. It took me too many years to realize that this researcher, the now late Karl Donath of Hamburg University, was right [11,12]. Karl Donath was a pioneer. As happens to many pioneers his work was forgotten when later American colleagues started discussing implants as foreign bodies well into our new millennium and Donaths’ papers published 15–20 years earlier were not even quoted. 

What is then an oral implant? Some colleagues of ours saw the implant as being the same as the tooth it was replacing, exemplified in this volume in the paper by Monje and co-workers [13]. In fact, such a coupling between the tooth and the implant once lead to the assumption that since teeth display a disease entitled periodontitis, then implants will display a similar disease that was named peri-implantitis. The original reason for the alleged disease was bacterial attack, even if, at least in the case of tooth disease, hereditary factors were also acknowledged. This is the background to seeing marginal bone loss around oral implants as solely a disease phenomenon. This outlook stands in clear contrast to orthopedic implants. In orthopedics marginal bone loss may be seen in a pattern similar to an oral implant. However, the reason for marginal bone loss in a hip arthroplasty is assumed to depend on a condition named aseptic loosening, i.e., something quite in contrast to what is believed with respect to oral implants. Aseptic loosening has in recent studies, one of them included in this volume [14], been shown to depend on immunological reactions. As summarized by Harris [15], massive immune reactions triggering osteoclasts may lead to bone resorption around hip arthroplasties. 

It would in fact seem very easy to conclude that the pathology of an oral implant is as little related to a tooth as is pathology of a hip arthroplasty to a normally functioning, pristine hip joint [16]. What then is behind the different opinion displayed by some dental colleagues? To my dismay, I must profess to clear guilt of the pioneering team of osseointegration of which I once was a member. When we worked to find out why we had seen oral implant success in clear contrast to all others who at the time had tried placing foreign devices in the oral cavity, we had several explanations. Among those were using minimal surgical trauma and commercially pure titanium implants that we at the time saw as being quite inert biologically, and presenting a simple wound healing phenomenon when placed in bone tissue [8,17]. Today we are aware of our misinterpretations. Trindade et al. [18,19] published two papers in this volume with evidence that titanium is not at all inert; the material causes clearly observable immune reactions in the tissue. Other biomaterials such as Poly-ether-ether-ketone, covered by Han et al. [20] in this volume, displayed significantly greater immune reactions than did titanium [19]. 

Another set of studies believed to prove that the presence of bacteria was the primary cause of problems with bone loss around implants relate to ligatures placed around experimental implants. One paper in this volume [21], summarized 133 such papers that generally reported a primary bacterial response to be behind the observed bone loss around the implants. However, in a ligature study conducted in a site where bacteria are usually absent, the tibia of research animals, Reinedahl and co-workers [22] reported of strong immunological reactions to the ligatures and subsequent marginal bone loss. Again, it seemed that the primary adverse reaction was immunological in nature and that bacteria were not needed for marginal bone loss which does not exclude a secondary bacterial action once the immune system has overreacted [23]. 

No, oral implants are not the same as a tooth. Neither does the primary bacterial theory explain why bone is lost around oral implants. We need a lot more research, such as several papers published in this volume of *Journal of Clinical Medicine*, to learn more about the true background of threats to osseointegration [24,25,26]. We must recognize that marginal bone loss commonly represents a complication to treatment; i.e., a condition, and not a disease. In addition, we can rejoice by the fact that moderately rough oral implant failure rates at 10 years of follow up are in the range of only 1–3% [27], that we see quite good clinical outcomes over 30 years of follow up [28] and that oral implants in case studies have been successfully followed up in excess of 50 years of clinical function [23]. 

Osseointegration is but an immunologically based reaction [29], representing demarcation of the foreign object [12], but if the immune system runs berserk the oral implant may be rejected from the body as a secondary response [12]. When the immune system in this manner overreacts and decides to reject the implant, at the same time the bacterial defense will go down, which explains the secondary presence of bacteria in failing implants [30].
